# Compliance and Determinants of Spectacle Wear Among Moroccan Adults Residing Beni-Mellal Khénifra Region

**DOI:** 10.22599/bioj.375

**Published:** 2024-10-18

**Authors:** Abderrahim Dahbi, Ahmed Chetoui, Farida Bentayeb, Samya Korziti, Abdelilah Errachidi, Youssef El Merabet

**Affiliations:** 1 Laboratory of Electronic Systems, Mechanical and Energy Information Processing, Faculty of Science, Ibn Tofail University, Morocco; 2 Biological Engineering Laboratory, Faculty of Sciences and Techniques, Sultan Moulay Slimane University, Mghila, PO. BOX: 523, 23000, Beni Mellal, Morocco

**Keywords:** Compliance, determinants, spectacle wearer, Visual impairment, Beni-Mellal Khenifra, Morocco

## Abstract

**Background and Objective:**

Ensuring a high level of adherence to wearing spectacles is essential to preserve eye health and achieve optimal vision correction. Comprehending the factors influencing adherence to wearing spectacles can inform strategies to improve eye care outcomes. This study aimed to assess the prevalence and factors influencing adherence to wearing spectacles among Moroccan adults residing the Beni-Mellal Khenifra region.

**Methods:**

A cross-sectional interview survey was conducted involving 389 adult spectacle wearers. Participants were recruited through a multilevel random sampling technique and interviewed using a structured questionnaire. This questionnaire collected data on demographics, spectacle prescription, usage patterns, and barriers to compliance. Compliance was assessed based on self-reported frequency and duration of spectacle wear according to the prescription. Possible factors influencing the wearing of spectacles were investigated using both univariate and multivariate regression models.

**Results:**

This study revealed that 57.6% of participants adhered to the recommended use of spectacles. Factors associated with compliance were higher education level, longer duration of spectacle use, awareness about the importance of spectacle wear, absence of a family history, specific refractive error types, and increased severity of refractive errors. Participants reported various reasons for not adhering to the recommended use of spectacles, with the most common being forgetfulness, perceiving improved vision without spectacle, discomfort while wearing spectacle, difficulty adapting to spectacle, and loss of spectacle.

**Conclusion:**

Given the high prevalence of non-compliance to spectacle wear among adults in the Beni-Mellal Khenifra region, it is evident that additional efforts are required to improve understanding and education regarding the advantages of consistent spectacle usage. Targeted educational and awareness initiatives have the potential to substantially enhance adherence rates and consequently improve visual health outcomes in the region.

## Introduction

Visual impairment represents a substantial public health concern, affecting millions of individuals and affecting their life quality and socioeconomic development (Jan et al., 2019; Lin and Yu, 2012). The World Health Organization (WHO) projects that over 2.2 billion people worldwide suffer from visual impairment or blindness, with a substantial proportion of these cases being preventable or treatable (WHO, 2019). In Morocco, it is estimated that approximately 2.2% of Morocco’s population had some form of visual impairment in 2019 (High Commission for Planning, 2020).

Refractive errors (RE) are a major cause of visual impairment and represent a critical public health issue globally. They are the second most common cause of blindness after cataracts and a leading cause of correctable visual disability (Bourne et al., 2013; WHO, 2023). The prevalence of uncorrected RE has increased over time, with approximately 123.7 million individuals worldwide now affected (WHO, 2023). When not addressed, RE can lead to social and economic challenges that affect both patients and their families (Hashemi et al., 2017; Naidoo and Jaggernath, 2012). Fortunately, these conditions are typically rectifiable through suitable vision correction means, including spectacles, contact lenses, or refractive surgery (Cochrane et al., 2010). Among these interventions, spectacles are commonly used to correct RE and visual impairments, including myopia, hyperopia, and astigmatism (Castanon Holguin et al., 2006). Proper spectacle wear is crucial not only for improving quality of life, but also for preventing further deterioration of eye health (Esteso et al., 2007). Despite the availability of corrective eyewear, compliance with spectacle wear recommendations varies among populations. Understanding the factors of influencing compliance is crucial for improving visual health outcomes. Several studies have identified biomedical, socio-demographic, and environmental factors influencing compliance with spectacle wear among adults (Congdon et al., 2008; Gogate et al., 2013; Li et al., 2010; Manny et al., 2012). Biomedical factors include the individual’s uncorrected visual acuity (UCVA), severity and nature of refractive errors (RE), improvement achieved through spectacles, and associated issues like headaches or eyestrain (Morjaria et al., 2019). Socio-demographic factors consist of age, gender, financial accessibility of spectacles, parental education levels, and psychosocial factors such as perception of wearing spectacle, comfort, aesthetic preferences, and concerns about appearance (Morjaria et al., 2019). Additionally, factors like lost or broken spectacles and environmental conditions can influence compliance with spectacle wear among adults. For example, individuals in dusty or hazardous environments may be less inclined to wear spectacles (Morjaria et al., 2019).

Understanding these multifaceted factors and addressing them appropriately are essential steps for healthcare professionals and spectacle manufacturers to promote better compliance among individuals with RE. This can therefore ensure that these individuals receive the vision correction they require for a healthier and more productive life.

In Morocco, as in many other countries, spectacles are a common means of vision correction. However, little is known about the level of compliance to spectacle wear and the factors that influence it among the Moroccan population. Hence, our study aims to investigate compliance with spectacles wear and identify associated factors among Moroccan participants from the Beni-Mellal Khenifra region. Understanding the factors that influence compliance with spectacles wear in this region will be beneficial for individuals involved in the design and implementation of local eye health programs.

## Methods

### Participants and study design

The present cross-sectional study was conducted in the Beni-Mellal Khenifra region of Morocco from July 15^th^ to September 5^th^, 2023. A total of 389 adult individuals who were required to wear vision spectacles were included in the study. They were selected through a multilevel random sampling technique.

The study’s sample size was calculated using the following formula:


\[
n\; = \;\frac{{{\mathrm{Z}^{2}}.\,{\mathrm{P}}(1 - {\mathrm{P}})}}{{E}^{2}}
\]

where P represents the estimated prevalence rate of compliance (50%). Given the absence of prior studies among adults in Morocco addressing our specific research question, with (E) representing a margin of error of 5% and (Z) representing the confidence level at 95% (with z = 1.96). This calculation yielded a minimum required sample size of 382 participants. To ensure accuracy and account for potential exclusions, we rounded this up to 400 participants.

The sample was drawn at three levels. First, we chose the five provinces of the region. Second, we selected an optical office at the level of each province. In total, five optical offices were chosen regionally. Third, for each optical office, a sample of 80 vision spectacle wearers meeting the inclusion criteria was recruited. On the day of the consultation, the first K participants to be recruited into the study who met the inclusion criteria was randomly selected by the researcher, and every K^th^ participant was recruited into the study thereafter. If the K^th^ person declined, the next person was invited. Recruitment continued until data were collected from 400 spectacle wearers.

After eliminating 11 questionnaires due to missing data or participants discontinuing the interviews prematurely, the final sample size was 389.

Inclusion criteria required participants to be 18 years or older and have a prescription for spectacle for vision correction. As spectacle compliance was self-reported through interviews, individuals aged less than 18 and those with mental disorders were excluded to mitigate potential response bias. Additionally, individuals physically unable to provide all data were also excluded from the study.

### Data collection

In this study, data were gathered through these one-to-one interviewsemploying a structured questionnaire.

The collected information included socio-demographics factors (such as age, gender, education level, and socioeconomic status), health insurance, spectacle prescription (type of vision problem, duration of spectacle usage, and regular eye check-ups), spectacle usage patterns (self-reported frequency and duration of spectacle wear), and barriers to compliance (challenges, discomfort, and discontinuation of spectacle use).

In this research, RE were evaluated using the spherical equivalent (SE), with the following criteria: mild ametropia (individuals with less than –3.00 diopters (D) of myopia or + 2 D of hyperopia), moderate ametropia (People with a RE falling between –3.00 to –6.00D for myopia or +2.00 to +6.00 for hyperopia), and severe ametropia (individuals surpassing –6.00 D for myopia or + 5 D for hyperopia.

The current study assessed compliance with spectacle wear based on participant self-report during interviews, categorizing participants as ‘non-compliant’ if they reported not consistently following the recommendations of their eye health professional regarding regular spectacle use.

Additionally, participants who did not wear their spectacles as recommended were requested to select a reason from a predefined list of possibilities derived from existing literature (Castanon Holguin et al., 2006; Congdon et al., 2008; Du et al., 2023; Morjaria et al., 2019).

### Data analysis

Statistical analysis was conducted using the Statistical Package for Social Sciences, version 25.0 (SPSS, Chicago, IL, USA) software. Continuous variables were expressed as mean ± standard deviation (SD), while categorical variables were represented as proportions. The compliance rate was reported in percentage terms and recorded in a binary format. Bivariate logistic regression was utilized to assess the statistical significance of the association between the dependent variable (spectacle non-compliance) and potential explanatory variables (participants’ sociodemographic characteristics and spectacle wear habits). Variables demonstrating a p-value < 0.05 in the bivariate logistic regression analysis were subsequently included in the multivariate logistic regression model to identify factors associated with spectacle compliance.

### Ethical considerations

The Ethical Committee for Biomedical Research of Oujda (CERBO), affiliated with the Faculty of Medicine and Pharmacy, University Mohammed Premier, Oujda, granted approval to this study (reference no. 51/2023). Before the interview, all participants were provided with thorough explanations regarding the study’s objectives, the significance of their involvement, and their right to refuse participation. Written consent was then obtained from each participant. The collected data underwent anonymization procedures to ensure the exclusion of any personally identifiable information.

## Results

### Demographic and spectacle-wearing characteristics of study participants

In this study, a total of 389 participants were considered, comprising 59.9% men and 40.1% women (Table 1). The age of the participants ranged from 18 to 72 years, with an average age of 40.57 ± 13.02 years. A total of 243 (62.5%) were married, and only 27 (6.9%) had no formal education.

**Table 1:** Socio-demographic characteristics of participants.


VARIABLES	CATEGORIES	N (%)/MEAN ± SD

Sex	Women	156 (40.1)

	Men	233 (59.9)

Age (y)	<30	92 (23.7)

	30–39	90 (23.1)

	40–49	110 (28.3)

	≥50	97(24.9)

Area of residence	Urban	288 (74)

	Rural	101 (26)

Marital status	Single	109 (28)

	Married	243(62.5)

	Divorced	24 (6.2)

	Widow/er	13(3.3)

Educational level	Illiterate	27 (6.9)

	Primary	80 (20.6)

	Secondary	155 (39.8)

	University	127 (32.7)

Health insurance	Yes	230 (59.1)

	No	159 (40.1)


Abbreviations: y: years; SD: standard deviation; n: number; %: percentage.

In our sample, the duration of wearing spectacles varied between 1 and 46 years, with an average of 10.90 ± 8.47 years. About 26% of the participants had been wearing spectacle for less than 5 years. Out of the 381 participants, 221 (56.8%) reported regular visits to a vision health professional as recommended. Additionally, a majority of participants (73%) had a family history of spectacle wear.

Among the respondents, 45.5% wore single spectacle, 12.1% exclusively used protection spectacle, and 17.2% wore near spectacle for reading or computer work, while the remaining 25.2% opted for progressive spectacle.

Regarding the prevalence of RE with a minimum of 0.50 D of spherical equivalent ametropia among participants, we observed that 44.7% of participants were affected by myopia, 39.3% were affected by hyperopia, and 15.9% wore spectacles for other reasons. Among the participants, 8.2% had severe myopia, while only 2.3% had severe hyperopia (Table 2).

**Table 2:** Spectacle wear characteristics of participants.


VARIABLES	CATEGORIES	N (%)/MEAN ± SD

Spectacles wear duration (y)	–	10.90 ± 8.47

Family history of spectacle wear	Yes	284(73)

	No	105(27)

Refractive errors type	Hyperopia	153 (39.3)

	Myopia	174 (44.7)

	Emmetropia (<0.5 D)	62 (15.9)

Severity of refractive errors	Mild myopia (<–3 D)	8.77 ± 2.53

	Moderate myopia (–3 D to –6 D)	(245) 30.6

	Severe myopia (>–6 D)	(556) 69.4

	Mild hyperopia (<+2 D)	116 (29.8)

	Moderate hyperopia (+2 D to +5 D)	28 (7.2)

	Severe hyperopia (+5.00 D)	9 (2.3)

	Normal (–0.5 to +0.5 D)	62 (15.9)

Spectacle type	Single vision	177 (45.5)

	Protection	47 (12.1)

	Near vision	67 (17.2)

	Progressive	98 (25.2)


Abbreviations: y: years; SD: standard deviation; n: number; %: percentage; D: diopter.

### Compliance to spectacle wear and its predictors

The results of this study revealed that 57.6% of participants were compliant with the recommended practice of wearing their spectacle regularly. In the bivariate regression analysis, several potential variables p < 0.05 were identified as significant factors affecting compliance with spectacle usage. These variables included level of education (p = 0.040), spectacle use duration (p < 0.001), family history of spectacle use (p < 0.001), receiving information about the importance of spectacle use (p < 0.001), type of RE (p < 0.001), and the severity of these RE (p < 0.003) (Table 3).

**Table 3:** Bivariate analysis of factors associated with compliance to spectacle wear among participants (n = 389).


CHARACTERISTICS		COMPLIANCE TO SPECTACLE WEAR		BIVARIATE ANALYSIS	
	
		YES n (%)	NO n (%)	ADJUSTED OR (95%CI)	*p*-VALUE

Gender	Female	81(51.9)	75(48.1)	1	

	Male	140(60.1)	93(39.9)	1.533(0.828–2.839)	0.174

Age (year)	<30	54(58.7)	38(41.3)		

	30–39	45(50)	45(50)		

	40–49	60(54.5)	50(45.5)	1.252(0.857–1.829)	0.246

	≥50	62(63.9)	35(36.1)		

Area of residence	Rural	54(53.5)	47(46.5)	1.104(0.566–2.153)	0.772

	Urban	167(58.0)	121(42.0)		

Marital status	Single	62(56.9)	47(43.1)		

	Married	147(60.5)	96(39.5)	1.268(0.872–1.845)	0.214

	Divorced	6(25)	18(75.0)		

	Widow/er	6(46.2)	7(53.8)		

Educational level	University	82 (64.6)	45(35.4)	1.333(1.013–1.754)	0.040**

	Secondary	95(61.3)	60(38.7)		

	Primary	38 (47.5)	42(52.5)		

	Illiterate	9(33.3)	18(66.7)		

Health Insurance	Yes	124 (53.9)	106(46.1)	1.015(0.516–1.994)	0.966

	No	97(61)	62(39)		

Spectacle wear duration (y)	<5	23(22.8)	78 (77.2)		

	5–9	54(47)	61(53)		

	10–14	44(74.6)	15(25.4)	2.245(1.815–2.777)	<0.001**

	15–19	49(81.7)	11(18.3)		

	≥20	51(94.4)	3(5.6)		

Family history of spectacle wear	Yes	157(55.3)	127(44.7)	2.728(1.393–5.342)	0.003**

	No	64(61)	41(39)		

Informed about spectacle wear	No	50(43.1)	66(56.9)	3.243(1.686–6.238)	<0.001**

	Yes	171(62.6)	102(37.4)		

Refractive errors type	Emmetropia	10(16.1)	53(83.9)	1.928(1.374–2.706)	<0.001**

	Myopia	137(78.7)	37(21.3)		

	hyperopia	77(50.3)	78(49.7)		

Severity of refractive errors	Mild	112(55.2)	91(44.8)	1.342(1.136–1.586)	0.001**

	Moderate	61(73.5)	22(26.5)		

	Severe	41(100)	0(0)		

Spectacle type	Single vision	133(75.1)	44(24.9)	1.150(0.880–1.501)	0.225

	Protection	6(12.8)	41(87.2)		

	Near vision	10(14.9)	57(85.1)		

	Progressive	72(73.5)	26(26.5)		


Abbreviations: y, years; SD, standard deviation; n, number; %, percentage; **: significant at P < 0.05.

The variables identified as significantly associated with compliance in the bivariate analysis were subsequently included in the multivariate analysis. In this multivariate analysis, using the same significance threshold (p < 0.05), several factors were confirmed to be significantly associated to a higher probability of adhering to spectacle usage (see Table 4). These factors include: high level of education (university, secondary), longer duration of spectacle use (10 years and above), having received information about the importance of spectacle wear, not having a family history of spectacle wear, having myopia and hyperopia, and increased severity of RE.

**Table 4:** Multivariate analysis of factors associated with compliance to spectacle wear among participants (n = 389).


CHARACTERISTICS		COMPLIANCE TO SPECTACLE WEAR		MULTIVARIATE ANALYSIS	
	
		YES n (%)	NO n (%)	ADJUSTED OR (95% CI)	*p*-VALUE

Educational level	University	82 (64.6)	45(35.4)	3.793(1.216–11.835)	0.022*

	Secondary	95(61.3)	60(38.7)	3.316(1.069–10.279	0.038*

	Primary	38 (47.5)	42(52.5)	1.798(0.542–5.963)	0.337

	Illiterate	9(33.3)	18(66.7)	1	

Spectacle wear duration (y)	<5	23(22.8)	78 (77.2)	1	

	5–9	54(47)	61(53)	1.502(0.754–2.994)	0.248

	10–14	44(74.6)	15(25.4)	6.887(2.932–16.175)	<0.001*

	15–19	49(81.7)	11(18.3)	7.797(3.009–20.205)	<0.001*

	≥20	51(94.4)	3(5.6)	34.066(8.689–133.56)	<0.001*

Family history of spectacle wear	Yes	157(55.3)	127(44.7)	1	

	No	64(61)	41(39)	2.070(1.105–3.877)	0.023*

Informed about spectacle wear	No	50(43.1)	66(56.9)	1	

	Yes	171(62.6)	102(37.4)	2.698(1.432–5.080)	0.002*

Refractive errors type	Emmetropia	10(16.1)	53(83.9)	1	

	Myopia	137(78.7)	37(21.3)	6.691(2.352–19.035)	<0.001*

	hyperopia	77(50.3)	78(49.7)	2.693(1.080–6.711)	0.034*

Severity of refractive errors	Mild	112(55.2)	91(44.8)	1	

	Moderate	61(73.5)	22(26.5)	2.933 (1.603–5.366)	0.001*

	Severe	41(100)	0(0)	-	0.001*


Abbreviations: y, years; SD, standard deviation; n, number; %, percentage; D, diopter; *, significant at p < 0.05.

### Most self-reported reasons for not adhering to spectacle usage

In the study, participants provided self-reported justifications for not complying with wearing spectacles. The predominant reasons included forgetting to wear spectacles, cited by 33.7% of participants, perceiving no improvement in vision with spectacles (30.1%), experiencing annoyance and discomfort while wearing spectacles (20.8%), and facing difficulties in adapting to spectacles (13.9%) (Table 5).

**Table 5:** Reasons reported by participants not complying with wearing spectaclesin descending order (n = 168).


SELF-REPORTED REASONS	PERCENTAGE (%)

Forgot spectacles	33.7

Perceived no improvement in vision with spectacles	30.1

Annoyance and discomfort	20.8

Difficulty adapting to spectacles	13.9

Spectacles lost	13.1

I don’t like spectacles	10.3

Additional impairment with spectacles	9.5

Concerned or teased about appearance of spectacles	9

Spectacles broken	5.9

Spectacles make me visually impaired	5.7

Cost of spectacles	4.9

Makes me dependent upon spectacles	3.3

Spectacles make my eyes grow inward	2.3

Do not perceive a necessity for wearing spectacles	1.5


## Discussion

The objective of the present study was to evaluate the prevalence and factors influencing adherence to wearing spectacles among individual residing the Beni-Mellal Khenifra region. The findings provide valuable insights into the determinants affecting adherence to spectacle usage within this population.

The findings revealed that just slightly over half of the participants adhered to the recommended practice of wearing spectacles. Moreover, participants of a higher level of education (university), having a longer duration of spectacle use, having received information about the importance of spectacle wear, without a family history of spectacle wear, having myopia and hyperopia, and with increased severity of RE were significantly associated with a higher compliance to wearing spectacles.

The compliance with spectacle wear in the investigated population was recorded at 57.6%. This figure is notably lower than the 87.4% reported in a study conducted in the USA (Kodjebacheva et al., 2011). Nevertheless, our recorded percentage surpasses the values reported in Mexico, which were at 13.4% for wearing spectacles (Castanon Holguin et al., 2006). Consistent with our findings, an examination of the literature regarding compliance with and spectacle wear predictors revealed that self-reported adherence levels varied between 58% and 82% (Morjaria et al., 2019).

In the multivariate logistic regression analysis, the attainments obtained revealed that compliance with spectacle wear showed no association with sex and age. The relationship between age and gender in relation to spectacle use has been inconsistently reported across different studies (Marmamula et al., 2014, 2009; Prema et al., 2008). However, it is noteworthy that other research studies have reported contrasting findings, indicating that males exhibited a lower propensity to wear spectacles compared to females (Congdon et al., 2008; Gogate et al., 2013; Keay et al., 2010). Similarly, other studies have observed a correlation between advancing age and a decreased tendency to use spectacles (Castanon Holguin et al., 2006; Pavithra et al., 2014). It is essential to consider that differences in the age and gender associations observed in our study compared to previous research may be explained by the inclusion criteria of participants in our sample. Previous studies have predominantly concentrated on spectacle compliance among children, and the shift to focusing on adults in our study may contribute to the observed variations.

The results from this study revealed that individuals with a higher educational attainment, specifically those with secondary (p = 0.038) and university (p = 0.022) education, demonstrated a greater tendency to comply with spectacle wear. Consistent with other studies, individuals with higher education levels exhibited increased odds of using spectacles (Marmamula et al., 2021; Marmamula et al., 2023). This association might be explained by considering the possibility that individuals with higher educational attainment may have faced increased visual demands, potentially due to extensive engagement in reading activities.

We also identified that the duration of spectacle use is an independent factor associated with compliance. This suggests that individuals who have been using spectacles for a longer time (10 years and above) are more likely to adhere to regular spectacle wear. The observed rise in adherence to prolonged spectacle use could be attributed to various potential explanations. Over time, wearing spectacles becomes a habitual behaviour for individuals who have used them for an extended duration, contributing to increased compliance. Additionally, individuals with a longer history of spectacle use may have experienced the positive effects of wearing spectacles, such as improved vision and comfort.

This research also found that individuals who were well informed about the significance of wearing spectacles seemed more likely to adhere to the recommendation. This finding aligns with a study conducted in Saudi Arabia (Aldebasi, 2011), which demonstrated that awareness of the significance of wearing spectacles increased compliance with spectacle use. Individuals who are well informed about the importance of wearing spectacles likely have a better understanding of the positive impact on their vision and overall eye health. This knowledge may lead to a greater appreciation of the benefits associated with consistent spectacle use.

Individuals without a family history of wearing spectacles were more inclined to adhere to guidelines. This aligns with a study conducted in the USA, which also demonstrated no association between the two factors (Sabharwal et al., 2021). Participants without a family history of spectacle wear may have a clearer understanding of their own visual needs. This awareness might lead to a stronger recognition of the importance of wearing spectacles for optimal vision, prompting greater compliance.

In terms of RE, both participants with myopia and hyperopia demonstrated higher compliance compared to emmetropic participants. Several studies have explored whether the type of refractive error affects spectacle wear. Consistent with our findings, a study conducted in India revealed differences among myopes, hyperopes, and emmetropes (Gogate et al., 2013). Interestingly, when comparing the results to studies conducted in Brazil and Oman (Khandekar et al., 2013; Yabumoto et al., 2009), there were no notable distinctions observed in spectacle compliance among individuals with varying types of RE. This suggests that cultural, regional, or other factors may play a role in shaping patterns of spectacle use, leading to varying results in different geographical locations (Khandekar et al., 2002; Yabumoto et al., 2009).

Our study demonstrated that, as the severity of RE increases, individuals in the study were more likely to adhere to prescribed or recommended interventions. This suggests that individuals with more severe vision issues perceive a greater need for corrective measures, leading to a higher adherence to prescribed interventions. It is noteworthy that this discovery aligns with findings from prior research (Du et al., 2023; Morjaria et al., 2019).

The results of the study provide valuable insights into the reasons for non-compliance with wearing spectacle among participants. The primary reason cited for noncompliance was forgetting to wear spectacles. Strategies such as creating reminders or establishing routines could potentially address this issue.

The second most reported reason perceived an improvement in vision without spectacles. Participants may feel that their vision is sufficiently clear without spectacles, leading to a perception that they are unnecessary. Educating participants about the long-term benefits of consistent wear, could be beneficial.

Other reported reasons included discomfort or annoyance with spectacles, which is a common cause of non-compliance. Difficulty adapting to spectacles, losing spectacles, and disliking the appearance of spectacles were also cited as contributing factors. Addressing comfort issues, providing support during the adaptation period, emphasizing the importance of keeping spectacles in a secure place, and offering a variety of frame options to suit individual preferences could be effective strategies to enhance compliance.

By highlighting these factors, this study brings attention to a relatively underexplored area in Morocco, characterized by limited available data. Moreover, it represents the inaugural study of its kind in the Beni-Mellal Khenifra region. Nevertheless, it is essential to acknowledge certain limitations in the survey, which should be taken into account when interpreting the study’s findings and their applicability to other populations or settings. Firstly, the cross-sectional design of the study offers only a snapshot of the participants’ compliance with spectacle wear at a single point in time. It cannot establish causality or long-term trends. Secondly, comparing our results with previous data proved challenging due to variations in methods for processing and reporting data across different recording time periods in various studies. Consequently, this situation hindered our ability to effectively compare the findings. Finally, compliance with spectacle wear was self-reported, which can introduce recall bias. Participants may overstate their compliance due to social desirability bias or underreport noncompliance.

## Conclusion

The study’s findings reveal that approximately 57% amongst adult spectacle wearers in the Beni-Mellal Khenifra region of Morocco were compliant with wearing their prescribed spectacles regularly. Several factors were identified as significant predictors of compliance, including a high level of education, longer duration of spectacle use (10 years and above), having received information about the importance of spectacle wear, not having a family history of spectacle wear, and specific refractive error types and severity.

Efforts to improve compliance should focus on the importance of education and awareness programs to promote the consistent use of spectacles, especially among individuals with high RE. Tailored interventions addressing common reasons for noncompliance, such as forgetfulness and discomfort, could significantly improve visual health outcomes in the Beni-Mellal Khenifra region of Morocco.

## References

[1] Aldebasi, Y. (2011) ‘Young public’s awareness to refractive error deficiency’, Int. J. Health Sci, 5, pp. 9–15. PMC3312764 22489225

[2] Bourne, R.R.A., Stevens, G.A., White, R.A., Smith, J.L., Flaxman, S.R., Price, H., Jonas, J.B., Keeffe, J., Leasher, J., Naidoo, K., Pesudovs, K., Resnikoff, S., Taylor, H.R. and Vision Loss Expert Group (2013) ‘Causes of vision loss worldwide, 1990–2010: a systematic analysis’, Lancet Glob. Health, 1, pp. e339–349. Available at: 10.1016/S2214-109X(13)70113-X 25104599

[3] Castanon Holguin, A.M., Congdon, N., Patel, N., Ratcliffe, A., Esteso, P., Toledo Flores, S., Gilbert, D., Pereyra Rito, M.A. and Munoz, B. (2006) ‘Factors associated with spectacle-wear compliance in school-aged Mexican children’, Invest. Ophthalmol. Vis. Sci, 47, pp. 925–928. Available at: 10.1167/iovs.05-0895 16505025

[4] Cochrane, G.M., du Toit, R. and Le Mesurier, R.T. (2010) ‘Management of refractive errors’, BMJ, 340, pp. c1711. Available at: 10.1136/bmj.c1711 20385718

[5] Congdon, N., Zheng, M., Sharma, A., Choi, K., Song, Y., Zhang, M., Wang, M., Zhou, Z., Li, L., Liu, X., Liu, X. and Lam, D.S.C. (2008) ‘Prevalence and determinants of spectacle nonwear among rural chinese secondary schoolchildren: the xichang pediatric refractive error study report 3’, Arch. Ophthalmol, 126, pp. 1717–1723. Available at: 10.1001/archopht.126.12.1717 19064854

[6] Du, K., Zhu, J., Guan, H., Zhang, Y., Wang, H., Wang, D. and Shi, Y. (2023) ‘Factors associated with the spectacle wear compliance among primary school students with refractive error in rural China’, Ophthalmic Epidemiol, 30, pp. 17–26. Available at: 10.1080/09286586.2022.2028295 35038950

[7] Esteso, P., Castanon, A., Toledo, S., Rito, M.A.P., Ervin, A., Wojciechowski, R. and Congdon, N.G. (2007) ‘Correction of moderate myopia is associated with improvement in self-reported visual functioning among Mexican school-aged children’, Invest. Ophthalmol. Vis. Sci, 48, pp. 4949–4954. Available at: 10.1167/iovs.07-0052 17962444

[8] Gogate, P., Mukhopadhyaya, D., Mahadik, A., Naduvilath, T.J., Sane, S., Shinde, A. and Holden, B. (2013) ‘Spectacle compliance amongst rural secondary school children in Pune district, India’, Indian J. Ophthalmol, 61, pp. 8–12. Available at: 10.4103/0301-4738.99996 23275214 PMC3555005

[9] Hashemi, H., Fotouhi, A., Yekta, A., Pakzad, R., Ostadimoghaddam, H. and Khabazkhoob, M. (2017) ‘Global and regional estimates of prevalence of refractive errors: Systematic review and meta-analysis’, J. Curr. Ophthalmol, 30, pp. 3–22. Available at: 10.1016/j.joco.2017.08.009 29564404 PMC5859285

[10] High Commission for Planning. (2020) Social indicators of Morocco 2019 (Les indicateurs sociaux du maroc 2019), Available at: https://www.hcp.ma.

[11] Jan, C., Xu, R., Luo, D., Xiong, X., Song, Y., Ma, J. and Stafford, R.S. (2019) ‘Association of Visual Impairment with Economic Development Among Chinese Schoolchildren’, JAMA Pediatr. 173, e190914. Available at: 10.1001/jamapediatrics.2019.0914 31058915 PMC6503578

[12] Keay, L., Zeng, Y., Munoz, B., He, M. and Friedman, D.S. (2010) ‘Predictors of early acceptance of free spectacles provided to junior high school students in China’, Arch. Ophthalmol. Chic. Ill, 1960 128, pp. 1328–1334. Available at: 10.1001/archophthalmol.2010.215 PMC4252047 20938003

[13] Khandekar, R., Gogri, U. and Al Harby, S. (2013) ‘The impact of spectacle wear compliance on the visual function related quality of life of Omani students: A historical cohort study’, Oman J. Ophthalmol, 6, pp. 199. Available at: 10.4103/0974-620X.122278 24379557 PMC3872572

[14] Khandekar, R., Mohammed, A.J. and Al Raisi, A. (2002) ‘Compliance of spectacle wear and its determinants among schoolchildren of Dhakhiliya region of Oman: A descriptive study’, SQU J. Sci. Res. Med. Sci, 4, pp. 39–43. PMC3174715 24019725

[15] Kodjebacheva, G., Brown, E.R., Estrada, L., Yu, F. and Coleman, A.L. (2011) ‘Uncorrected refractive error among first-grade students of different racial/ethnic groups in southern California: results a year after school-mandated vision screening’, J. Public Health Manag. Pract, 17, pp. 499–505. Available at: 10.1097/PHH.0b013e3182113891 21964359

[16] Li, L., Lam, J., Lu, Y., Ye, Y., Lam, D.S.C., Gao, Y., Sharma, A., Zhang, M., Griffiths, S. and Congdon, N. (2010) ‘Attitudes of students, parents, and teachers toward glasses use in rural China’, Arch. Ophthalmol, 128, pp. 759–765. Available at: 10.1001/archophthalmol.2010.73 20547954

[17] Lin, J.-C. and Yu, J.-H. (2012) ‘Assessment of quality of life among Taiwanese patients with visual impairment’, J. Formos. Med. Assoc. 111, pp. 572–579. Available at: 10.1016/j.jfma.2011.09.021 23089693

[18] Manny, R.E., Sinnott, L.T., Jones-Jordan, L.A., Messer, D., Twelker, J.D., Cotter, S.A., Kleinstein, R.N., Crescioni, M. and Group, T.C.S. (2012) ‘Predictors of adequate correction following vision screening failure’, Optom. Vis. Sci, 89, pp. 892. Available at: 10.1097/OPX.0b013e318255da73 22544001 PMC3365655

[19] Marmamula, S., Barrenkala, N.R., Khanna, R.C., Challa, R., Bhakki, M., Kumbham, T.R., Modepalli, S.B., Yellapragada, R. and Friedman, D.S. (2021) ‘Near vision impairment among the elderly in residential care-the Hyderabad Ocular Morbidity in Elderly Study (HOMES)’, Eye Lond. Engl, 35, pp. 2310–2315. Available at: 10.1038/s41433-020-01243-w PMC8302663 33159176

[20] Marmamula, S., Bhoopalan, D., Kumbham, T.R., Yelagondula, V.K. and Keeffe, J. (2023) ‘Prevalence, pattern, and compliance with spectacles use among the elderly in homes for the aged in South India: The Hyderabad Ocular Morbidity in Elderly Study (HOMES)’, Indian J. Ophthalmol, 71, pp. 263–267. Available at: 10.4103/ijo.IJO_884_22 36588247 PMC10155529

[21] Marmamula, S., Keeffe, J.E. and Rao, G.N. (2009) ‘Uncorrected refractive errors, presbyopia and spectacle coverage: results from a rapid assessment of refractive error survey’, Ophthalmic Epidemiol, 16, pp. 269–274. Available at: 10.1080/09286580903144720 19874105

[22] Marmamula, S., Khanna, R.C., Narsaiah, S., Shekhar, K. and Rao, G.N. (2014) ‘Prevalence of spectacles use in Andhra Pradesh, India: rapid assessment of visual impairment project’, Clin. Experiment. Ophthalmol, 42, pp. 227–234. Available at: 10.1111/ceo.12160 23845055

[23] Morjaria, P., McCormick, I. and Gilbert, C. (2019) ‘Compliance and predictors of spectacle wear in schoolchildren and reasons for non-wear: a review of the literature’, Ophthalmic Epidemiol, 26, pp. 367–377. Available at: 10.1080/09286586.2019.1628282 31181970

[24] Naidoo, K.S. and Jaggernath, J. (2012) ‘Uncorrected refractive errors’, Indian J. Ophthalmol, 60, pp. 432–437. Available at: 10.4103/0301-4738.100543 22944755 PMC3491271

[25] Pavithra, M., Hamsa, L. and Madhukumar, S. (2014) ‘Factors associated with spectacle-wear compliance among school children of 7–15 years in South India’, Int. J. Med. Public Health, 4, pp. 146. Available at: 10.4103/2230-8598.133110

[26] Prema, R., George, R., Sathyamangalam Ve, R., Hemamalini, A., Baskaran, M., Kumaramanickavel, G., Catherine, M. and Vijaya, L. (2008) ‘Comparison of refractive errors and factors associated with spectacle use in a rural and urban South Indian population’, Indian J. Ophthalmol, 56, pp. 139–144. Available at: 10.4103/0301-4738.39119 18292625 PMC2636078

[27] Sabharwal, S., Nakayoshi, A., Lees, C.R., Perez, S. and De Alba Campomanes, A.G. (2021) ‘Prevalence and Factors Associated With Eyeglass Wear Compliance Among Preschoolers From Low-Income Families in San Francisco, California’, JAMA Ophthalmol, 139, pp. 433. Available at: 10.1001/jamaophthalmol.2020.7053 33599687 PMC7893547

[28] World Health Organization. (2019) ‘World report on vision’ ISBN 978-92-4-151657-0, Geneva: World Health Organization. Available at: https://www.who.int/publications/i/item/9789241516570 (accessed 19 Decembre 2023).

[29] Word Health Organization. (2023) Vision impairment and blindness [WWW Document]. Available at: https://www.who.int/news-room/fact-sheets/detail/blindness-and-visual-impairment (accessed 12.19.23).

[30] Yabumoto, C., Hopker, L.M., Daguano, C.R., Basilio, F.M.A., Robl, R., Rodrigues, D.B., Jimenez, A., Moreira, A.T.R., Sakata, L.M. and Sakata, K. (2009) ‘Factors associated with spectacles-use compliance in a visual screening program for children from southern Brazil’, Invest. Ophthalmol. Vis. Sci, 50, pp. 2439.

